# Detection of virulence factors in opportunistic bacteria: advances, challenges, and practical implementation

**DOI:** 10.3389/fmicb.2025.1638925

**Published:** 2025-09-17

**Authors:** Igor Chebotar, Ilya Azizov, Mikhail Edelstein, Roman Kozlov

**Affiliations:** Institute of Antimicrobial Chemotherapy, Smolensk State Medical University, Smolensk, Russia

**Keywords:** bacterial infection, human opportunistic pathogen, virulence mechanisms, detection of virulence factors, bacterial fitness

## Abstract

Virulence is a property of bacteria that determines the degree of damage inflicted on humans. In modern medicine, there is an underestimation of the importance of testing the virulence of opportunistic pathogens to assess prognosis, optimize therapy, and evaluate the risk of developing probable complications of the infectious process. This review analyzes the basic characteristics of virulence, including multifactoriality, complex regulation, its relationship with fitness and bet-hedging in the context of choosing optimal methods for quantitative virulence testing. The possibilities of various methodological approaches for evaluation of virulence in clinical laboratory settings are considered. Current technology levels allow laboratories to be equipped with test systems for implementing methods to detect the virulence of clinical opportunistic isolates. At the hospital level, determining the virulence of isolates from individual patients will improve the prediction of the course of the infectious process and help to rationalize infection control based on principles similar to those used in combating antibiotic-resistant strains. Knowledge of virulence properties of relevant pathogen clones is required for the successful development of anti-virulence strategies.

## Introduction

The global damage caused by bacterial infections results in a staggering loss of life. Each year, approximately 7.7 million people die from bacterial infections worldwide (bacteria-attributable deaths), over 3.7 million people die from respiratory infections and tuberculosis (2017) (Okeke et al., 2024; Naghavi et al., 2024). The dynamic symbiotic relationship between a microbe and its host during an infection is commonly described as an infectious process. The outcome of each infectious process depends on the interaction of three components: (1) the host with its immune defense, (2) the microbe, whose strategy is aimed at multiplying within the host organism while evading and manipulating immune effectors, (3) external medicinal interventions designed to suppress the microbe and protect the host. The microbe’s tactics in the context of infectious diseases often accompanied direct or indirect (immune-mediated) damaging the host tissues. To describe a microbe’s ability to cause harm to the host, the term virulence is employed. The concept of “virulent” began to be actively used in medieval medicine (from about 1380 A.D.) to describe ulcers that emitted poison (Lanfrank, 1894). Later, virulence came to denote toxicity and contagiousness (Simpson, 1665; Wynell, 1670), eventually becoming synonymous with plant toxicity and chemical compounds (Kircheri, 1665). Before Louis Pasteur’s revolutionary discovery, scientists believed that infections were caused by substances such as “morbid poisons,” that “arise from disease, and excite a secretion capable of reproducing the same disease” (Adams, 1807). Even then, to describe the degree of danger posed by these “morbid poisons,” the epithet “virulent” was applied, and attempts were made to destroy their “virulence” (of smallpox, gonorrheal matter) using available chemicals (“chlorine gas,” iodine, etc.), (Browne, 1830). Naturally, the term virulence became associated with newly discovered bacteria causing infections. As early as Pasteur (1881, 1882) widely used the term virulence in relation to pathogenic microbes, understanding it as the degree of danger. Both Pasteur (1881, 1882), Koch et al. (1884) used the term virulence, attributing quantitative characteristics to it. Koch et al. (1884), describing experiments to develop a vaccine against anthrax, confirmed the possibility of reducing the virulence of the “anthrax virus” through cultivation “under certain conditions” (Koch et al., 1884). Today, the meaning of the term virulence essentially corresponds to how it was understood by the pioneers of microbiology - the degree of the injury-producing potential or toxicity of a microorganism. During Koch (1884), Pasteur’s (1881, 1882) time, the criterion for assessing the virulence of a pathogen was its ability to cause disease in animals or humans. This property formed the basis of the classical Henle-Koch postulates, which work well for microbes classified as pathogens. Non-opportunistic pathogens are microorganisms that cause disease in practically healthy persons with high probability (definitions of key terms used in this review are summarized in Supplementary Table 1). For many non-opportunistic pathogens (*Bacillus anthracis*, *Vibrio cholerae*, *Burkholderia mallei*, etc.,) species identification alone is sufficient for accurately predicting their degree of danger. Sometimes, however, additional confirmation of virulence is required, such as evaluating the toxigenicity of a strain, as in the case of *Corynebacterium diphtheriae*. Overall, assessing virulence of non-opportunistic today does not raise significant questions. Standard protocols exist for such bacteria, allowing precise predictions of their danger and enabling effective control measures.

When dealing with opportunistic pathogens, the situation becomes more complicated. Virulence manifests itself in a dialectical interplay between the host and the microorganism(s). One perspective holds that microbial virulence should be viewed in the context of its interaction with the host (Casadevall and Pirofski, 2015). Indeed, immune effectors can influence virulence levels via up- or down-regulation (Zhou et al., 2023), as well as through mutation-driven processes leading to the emergence of clones with novel virulence profiles (Rossi et al., 2021). This confusion profoundly complicates our understanding of virulence mechanisms and hampers the development of effective evaluation methods. Following the principles of cognition outlined by René Descartes, the complexity of any phenomenon requires its analysis through decomposition into constituent components. In this review, we focus solely on attributes of virulence in opportunistic bacteria, specifically highlighting opportunities and benefits associated with their evaluation.

The material foundation of pathogen virulence lies in virulence factors. A virulence factor is a component of a pathogen that contributes to the progression of the infectious process. Deletion of the virulence factor may compromise virulence but not viability (Casadevall and Pirofski, 1999). Virulence factors can encompass modulins affecting host immune responses (Henderson et al., 1996). The cumulative virulence of a pathogen depends on the integral functioning of its virulence factors. Therefore, assessing virulence factors serves as a vital basis (alongside antibiotic resistance and host condition) for determining pathogen risk. Evaluating virulence in opportunistic pathogens involves numerous uncertainties. Key questions include (1) the specificity of opportunistic pathogens’ virulence manifestations depending on the form of the disease, (2) the feasibility of clinical prediction based on the assessment of the virulence of a specific isolate, (3) the epidemiological threat posed by strains with increased virulence, and (4) the value of information about virulent isolates for optimization of infection control. Answering these questions requires verifying data on the correlation between disease outcomes and virulence profiles of causative agents, as well as accumulating information on the distribution of different pathovars among opportunistic pathogens. To gather such information, species-specific methodologies for virulence evaluation must be defined. While this review does not provide ready-made detailed solutions, it discusses the theoretical foundation for developing virulence assessment methods and analyzes and systematizes the most relevant examples of determining the virulence of current opportunistic pathogens. Modern technological capabilities for detecting virulence using accessible, reproducible, and standardized methods will also be justified.

The main reasons for the complexity of assessing the virulence of opportunistic pathogens can be attributed to (1) difficulties in defining correlations between opportunistic bacteria’s virulence factors and the manifestation of the infectious process, (2) the multifactorial nature of virulence, (3) the complex and unclear regulation of virulence expression, and (4) challenges in choosing appropriate methods for assessing virulence.

## Locus-specific virulence factors

Typically, major opportunistic bacteria exhibit diversity in loci and forms of infection, affecting various organs and systems and causing infections of varying severity, acuteness, and duration. This raises the question of whether different virulence factors play equally important roles in realizing pathogenicity across different loci. Such patterns have been observed in some species. For example, in *Pseudomonas aeruginosa* in chronic ankle wounds, T3SS, responsible for secreting contact toxins like ExoU, ExoS, ExoT, and ExoY, is inhibited (Karash et al., 2021). In cystic fibrosis, *exoS*(+)/*exoU*(+) virulotypes often cause chronic forms of cystic fibrosis pneumonia, whereas *exoS*(+)/*exoU*(−) isolates are responsible for early clinical lung alterations (Sarges et al., 2020; Savinova et al., 2020). Despite statistically proven correlations, attention should be paid to the absence of absolute rules governing the distribution of virulence genes among isolates obtained from different loci. All results showed numerous exceptions. Traditionally, it has been believed that uropathogenic *Escherichia coli* strains primarily carry specific adhesins, including type 1 fimbriae, P fimbriae, S fimbriae, F1C fimbriae, and afimbrial adhesin (Sarowska et al., 2019). It is considered that the primary attribute of *E. coli* strains causing neonatal meningitis is a set of factors, chiefly the K1 capsule, which protects them from immune effectors and is rarely found in uropathogenic strains (Sarowska et al., 2019). Overall, the information on the virulent features of extraintestinal pathogenic *E. coli* (ExPEC) is enormous and often contradictory. However, some conclusions can be drawn today. Firstly, there are no single molecular/genetic virulence factors that could be unique ExPEC indicators similar to such markers of virulence (toxigenicity) as diphtheria toxin in *Corynebacterium diphtheriae* or Shiga-toxin in *Shigella dysenteriae* (Johnson and Russo, 2018). Secondly, differences between ExPEC and commensal *E. coli* strains lie in complex combinations of true virulence genes and regulatory genes, possibly also in their expression patterns. Emphasizing this point, Russo and Johnson (2000) highlighted the c but rather “various combinations of adhesins (e.g., P and S fimbriae), iron-acquisition systems (e.g., aerobactin), host defense-avoidance mechanisms, and toxins (e.g., hemolysin), which collectively are now regarded as extraintestinal virulence factors.” Commensal strains typically lack specialized virulence traits found in ExPEC. Additionally, ExPEC and commensal *E. coli* differ in their accessory genomes. Genomic studies have shown the presence of horizontally acquired pathogenicity-associated islands more frequently observed in ExPEC than in commensals (Johnson and Russo, 2018). The more frequent association of ExPEC pathotypes with phylogroup B2 also indicates the specificity of their virulent ensembles (Hazen et al., 2023). Although some specific virulence factors such as adhesins, iron-acquisition systems, toxins, and other “can distinguish ExPEC from typical commensal *E. coli*, comparative genomics has revealed that this distinction is far from absolute” (Russo and Johnson, 2000).

For other opportunistic pathogens, the correlation between the presence of pathogenicity factors and preferred infection sites remains even less clear. While various intoxications attributed to *Staphylococcus aureus* are strictly linked to specific toxins, opinions regarding the locus-specificity of virulence factors in *S. aureus* infections are quite contradictory. In the study by Budzyńska et al. (2021), it was found that *sea-* and *sei-*toxin genes were more frequent among blood strains than in wound isolates. Although the difference was statistically significant, it did not support any absolute locus-specificity of toxin genes: the *sea*-gene was present in 6.0% of blood strains and 19.0% of wound strains, while the *sei*-gene was found in 46.0% of blood strains and 61.0% of wound strains. The exfoliative toxin *eta* gene was detected in 14% of wound strains and 13% of blood strains, the toxic shock toxin (*tst*) gene was found in 13% of wound strains and 9% of blood strains, and the Panton-Valentine leukocidin gene (*lukSF-PV*) was identified in 3% of wound strains and 1% of blood strains (Budzyńska et al., 2021; Dermota et al., 2024; Gu et al., 2024).

Probably in the future, a detailed deciphering of the mechanisms of pathogenesis and accumulation of information on correlations between the virulence profile of the pathogen and the variant of infection will help obtain a clearer picture of the locus-specificity of various virulence factors.

A practically significant question arises: is it necessary, when detecting virulence in clinical isolates, to pay attention to the phenomenon of locus-specificity and determine only those factors that have predictive significance for a particular type of infection? If we focus solely on the individual patient, it would suffice to identify only the prognostically significant factors for the locus of infection. However, if we think strategically from the perspective of infection control and surveillance tasks, it becomes essential to obtain information about the most critical virulence factors of each isolate. This necessity follows from the existence of cross-pathovars (Lindstedt et al., 2018), which may pose nosocomial epidemic threats. It is likely prudent to follow a strategic course and test all key factors that could yield strategically valuable information about the virulence profiles of pathogens.

## Multifactorial nature of virulence in opportunistic pathogens and key virulence factors

Any pathogenic or opportunistic bacterium realizes its virulence during infection by engaging multiple factors. There are no exceptions. Even pathogens causing mono-intoxications require multiple tools to implement the infectious process. For example, typical diphtheria caused by *Corynebacterium diphtheriae* is determined by the specific diphtheria toxin. However, clinically evident diphtheria would be impossible without the initial stages of infection, including preliminary colonization of mucosal surfaces (or less commonly wound surfaces) by *C. diphtheriae* and bacterial survival under conditions of localized inflammation. To realize these initial stages, *C. diphtheriae* expresses a variety of factors, including diverse adhesins, iron uptake systems, cytotoxic proteins, apoptotic and inflammatory inducers (Rogers et al., 2011; Cerdeno-Tarraga et al., 2003; Weerasekera et al., 2019; Ott et al., 2022). According to the Virulence Factor Database (VFDB), the virulome of uropathogenic *E. coli* includes > 90 genes, *Acinetobacter baumannii* complex > 110 genes, *P. aeruginosa* > 260 genes, *Klebsiella pneumoniae* > 120 genes, *S. aureus* > 110 genes, and *Enterococcus spp.* > 40 genes (The Virulence Factor Database, 2025). The virulome is the set of genes that contribute to the virulence of a pathogen, regardless of the significance of each of these genes. Thus, every product of the virulome can contribute directly or indirectly to the overall virulent potential of the pathogen. However, the pathogenetic significance of various virulence factors is not the same. The concept of virulence genes includes three distinct types of genes including (1) true virulence genes, that are directly involved in causing disease, (2) virulence-associated genes that encode regulators/activators of true virulence genes, (3) virulent life-style genes that ensure viability in the host organism (Wassenaar and Gaastra, 2001). The large volume of information about the virulome does not guarantee an understanding of the significance of each virulence gene in the realization of the infectious process. This difficulty arises due to the inability to predict precisely the expression of each gene, which is determined by complex signals from the external environment and enigmatic regulatory mechanisms within the bacterial cell. Redundancy lies in the fact that identifying the entire virulome provides much information already known to the species and represented by constitutively expressed species-specific genes.

**FIGURE 1:**
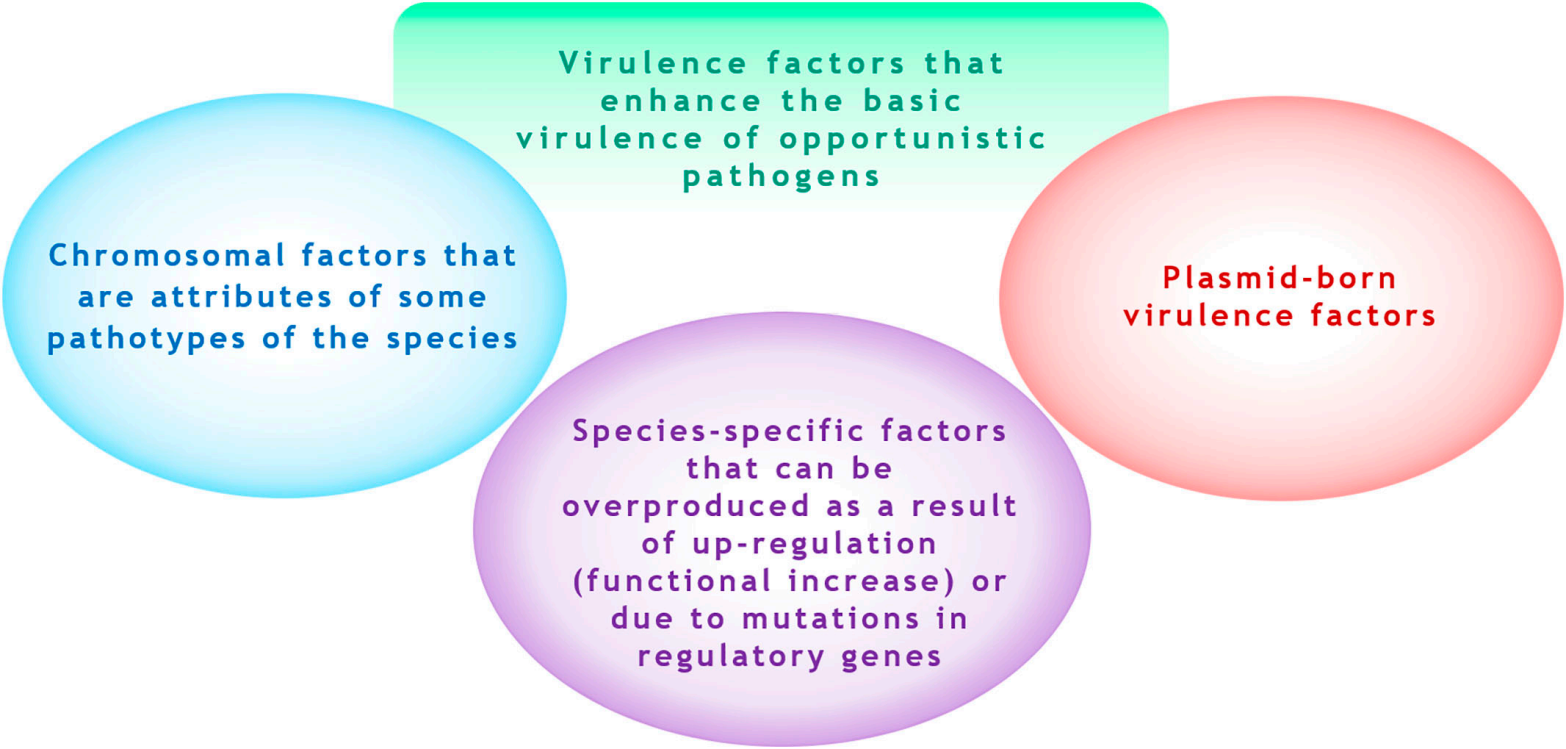
Virulence factors that enhance the basic virulence of opportunistic microorganisms.

This suggests that it is rational only to identify pathogenetic significant factor, which can “enhance” the baseline natural level of virulence of the species. We refer to these as acquired virulence factors. These factors can include (1) chromosomal (non-plasmid-borne) factors that may be overproduced due to “up-regulation” of expression (functional increase either because of mutations in regulator genes), and (2) plasmid-borne factors (Figure 1). A striking example of a species-specific overexpressed factor is the hyperproduction of the alginate by *P. aeruginosa* isolates due to mutational inactivation of the *mucA* gene or resulting from up-regulation via activation of the transcriptional regulator σ^22^ by environmental signals (Boucher et al., 1997; Damron and Goldberg, 2012). Examples of plasmid-borne factors include siderophores aerobactin and salmochelin and the determinant of hypermucoidy (*rmpA*) in *K. pneumoniae* (Lam et al., 2018).

The contribution of each acquired factor to the overall portrait of virulence is incomparable. To reduce the list of tested virulence factors, emphasis should be placed exclusively on key acquired factors. By key virulence factors, we mean virulence factors without which opportunistic pathogens become incapable of causing the disease or result in mild forms of the disease. The concept of the key virulence factor can include both factors encoded by a single gene and factors whose production may depend on a set of different genes. In other words, a key virulence factor must comply with Falkow (2004) rule, which states: “Specific inactivation of the gene(s) associated with the suspected virulence trait should lead to a measurable loss in pathogenicity or virulence… Reversion or allelic replacement of the mutated gene should lead to restoration of pathogenicity….” The most accurate interpretation of the mechanism of action of a virulence factor on host structures should additionally be validated in experiments using animal models knocked out of the virulence factor target gene.

One approach to evaluate virulence involves counting the number of pathogenicity factors in an isolate using phenotypic or genetic methods. One option for such counting is the virulence index, that is defined as the ratio between positive tests for virulence traits and the total amount of virulence traits evaluated and calculated for each isolate (Singh et al., 2017; Cunha et al., 2023). This method has been applied to identify virulence factors without validating their pathogenic significance, such as bacterial hydrophobicity (Cunha et al., 2023). Furthermore, verified examples demonstrating its reliability for predicting infection outcomes in humans are currently unavailable. Nonetheless, we should not overlook the underlying principle of this approach, grounded in quantitative virulence assessment, which could benefit from being complemented by ranking based on expression levels and significance.

Another measure of virulence is the virulence score, which is calculated as the sum of virulence factor genes in each isolate. It should be noted that the virulence score is not a standardized indicator. Different authors calculate it differently, even when analyzing the virulence of the same species. In studies on *K. pneumoniae*, some authors combine all genes involved in virulence into the virulence score (Maldonado et al., 2024), others count only siderophore genes (Lam et al., 2021), and still others add up genes encoding adhesins, capsules, iron uptake systems, invasins, and toxins (Hsiao et al., 2024). The popular Kleborate software calculates the virulence score for *K. pneumoniae*, considering virulence loci, including YbST (yersiniabactin), CbST (colibactin), AbST (aerobactin), SmST (salmochelin), and RmpA/RmpA2 (hypermucoidy) genes (Lam et al., 2021). The cited articles do not involve quantitative assessment of virulence factor production, which constitutes their limitation. Other disadvantages of using virulence scoring will be addressed later. Nevertheless, calculating both the virulence score and the virulence index represents reasonable efforts to prioritize the most important virulence factors.

Hypothetically, the existence of key factors can be determined for all nosocomial pathogens, including bacteria with poorly studied virulome. For example, the inactivation of T3SS with the AxoU toxin in *Achromobacter spp.*, predicted merely as an ortholog of the *P. aeruginosa* ExoU toxin, leads to a reduction in the severity of infection and transforms acute forms into chronic ones (Tessmer et al., 2019; Pickrum et al., 2020). This indicates that the AxoU toxin is a key factor for *Achromobacter spp*. If key factors cannot be identified in a pathogenic bacterium, this does not imply their absence; rather, it reflects a lack of knowledge about their virulome and how they realize their virulent potential at different stages of infection.

Selecting key factors worth testing is challenging. Given the contradictions in correlations between locus-specificity of the infectious process and the presence of virulence factors in many pathogens (see above), the objects of testing should be key factors whose role in pathogenesis is undeniable. For major opportunists, key virulence factors are well-known. For example, in bloodstream infections, *P. aeruginosa* isolates actively expressing ExoU are more likely to result in poor outcomes regardless of their antibiotic susceptibility profile (El-Solh et al., 2012; Peña et al., 2015). In cases of septic shock, high mortality rates in *P. aeruginosa* have been associated with non-motile or pyocyanin hyperproducing isolates (Gupte et al., 2021).

We attempted to present promising acquired key virulence factors or their markers for testing in some opportunistic bacteria in Table 1. The selection of bacteria was based on their relevance, including opportunistic species belonging to the ESKAPE group of pathogens, except *Enterobacter* spp., as well as extraintestinal pathotypes of *E. coli*. The criteria for inclusion of virulence factors in the table were (1) confirmed *in vivo* (experimentally or based on clinical isolate research) pathogenetic significance, including cases of hyperproduction, and (2) variability (qualitative and/or quantitative) in factor production. The exclusion criteria included (1) the presence of the factor in the overwhelming majority of commensal/environmental isolates or clinical isolates from patients with mild, non-invasive, and non-recurrent forms of infection in the absence of hyperproduction in severe forms of infection, and (2) the lack of proper verification of the pathogenetic significance of the virulence factor or its overproduction, verified experimentally *in vivo* or based on clinical isolate research. The list of virulence factors presented in Table 1 is neither final nor fully verified. Only additional information derived from extensive virulence testing can enhance it.

**TABLE 1:** Promising virulence factors or their markers for testing important opportunistic pathogens.

Bacterium	Virulence factor			
	Structure (main associated gene)	Detection*	Function	
*A. baumannii*	Capsule: KL2, KL3/KL22, 17 KL, and KL49	Genome sequencing, PCR (Wyres et al., 2020)	Immune effector evasion	Jacobs et al., 2010; Sato et al., 2019; Russo et al., 2010; Ahmad et al., 2023; Yang et al., 2022; Rakovitsky et al., 2021; Cahill et al., 2022; Cook-Libin et al., 2022; Sheldon and Skaar, 2020; Deng et al., 2020; Singh et al., 2019; Conde-Pérez et al., 2021; Yang et al., 2019; Christian et al., 2018
	Biofilm formation (*bap* and *ompA, scu-*genes)	Christensen’s tube test, biofilm ring test, microtiter plate method, the Calgary biofilm device (Christensen et al., 1982; Olivares et al., 2016; Peeters et al., 2008; Ceri et al., 1999)	Immune evasion and antibiotic resistance	
	Phospholipase D hyperproduction (*pld*)	The Amplex Red Phospholipase D Assay Kit (Jacobs et al., 2010)	Cytotoxicity	
	Acinetobactin (*bas*-genes)	CAS-test (universal siderophore test), PCR (Schwyn and Neilands, 1987; Mihara et al., 2004)	Iron uptake (siderophore)	
	Mucoid factor, hypermucoviscosity (*wzc* overexpression)	String-test, India ink staining, Congo red phenotypic test, genome/transcriptome sequencing, density-dependent gradient test, centrifugation resistance test (Rakovitsky et al., 2023; Haddad et al., 2018; Shan et al., 2021; Kon et al., 2020; Sattler et al., 2024)	Immune effector evasion	
	Catalase, hyperproduction (*katE/G*)	The Catalase Colorimetric Activity Kit (Sato et al., 2019)	Survival within phagocytes	
*E. coli* (extraintestinal strains)	YfC fimbria (*yfcV*)	PCR assay (Spurbeck et al., 2012)	Adhesin	Johnson et al., 2018; Peerayeh et al., 2018; Guo et al., 2024; Arisoy et al., 2006; Garcia et al., 2011; Schmidt and Hensel, 2004; Hung et al., 2019; Lara et al., 2017; Hemati et al., 2024; Díaz et al., 2020
	P fimbriae (*papG*)	PCR assay (Johnson and Stell, 2000)	Adhesin	
	S fimbriae (*sfa*)	PCR assay (Davari Abad et al., 2019)	Adhesin	
	Aerobactin (*aer* or *iucC*)	CAS-test (universal siderophore test), multiplex PCR assay (Schwyn and Neilands, 1987; Searle et al., 2015)	Iron uptake (siderophore), pathogenic island marker	
	Yersiniabactin (*ybt*)	CAS-test (universal siderophore test), multiplex PCR assay (Schwyn and Neilands, 1987; Searle et al., 2015)	Iron uptake (siderophore)	
	Vacuolating autotransporter toxin (*vat*)	PCR assay (Nichols et al., 2016)	Cytotoxicity	
	Protein IbeA (*ibeA*)	PCR assay (Johnson and Stell, 2000)	Significant invasin in neonatal meningitis *E. coli* strains	
	Enzyme II of the phosphotransferase system (*malX*)	PCR assay (Adwan et al., 2016)	Pathogenic island marker	
*K. pneumoniae*	Capsule: K-1, K-2, K-5, K-20, K54, and K57 (*wzy* and *wzx*)	PCR (Hasani et al., 2020; Turton et al., 2010)	Immune effector evasion	Stanton and Wyres, 2024; El Fertas-Aissani et al., 2013; Turton et al., 2008; Russo et al., 2015; Huang et al., 2022; Li and Ni, 2023; Bulger et al., 2017
	Yersiniabactin (*ybt*)	CAS-test (universal siderophore test), PCR (Schwyn and Neilands, 1987; Bachman et al., 2011)	Iron uptake (siderophores)	
	Aerobactin (*iuc*)	CAS-test (universal siderophore test) (Schwyn and Neilands, 1987), PCR (Bachman et al., 2011), biological assay for aerobactin production (Carbonetti and Williams, 1984)	Iron uptake (siderophores)	
	Mucoid factor, hypermucoviscosity (*rmpA/rmpA2*)	Congo red phenotypic test (Haddad et al., 2018), string-test, PCR (Mai et al., 2023), centrifugation resistance test (Sattler et al., 2024)	Immune effector evasion	
	Biofilm formation (*pgaABCD*)	Christensen’s tube test (Christensen et al., 1982), biofilm ring test (Maldonado et al., 2024), microtiter plate method (Peeters et al., 2008), Calgary biofilm device (Ceri et al., 1999)	Immune effector evasion and antibiotic resistance	
	Metabolite Transporter PEG344 (*peg-344*)	PCR (Russo et al., 2018)	Hypervirulence indicator with unclear function	
*P. aeruginosa*	Exotoxin A (ExoA)	ELISA (Shigematsu et al., 2007)	Cytotoxicity	Sánchez-Diener et al., 2020; Miyazaki et al., 1995; May et al., 1991; Jeong et al., 2024; Tahmasebi et al., 2022; Hauser et al., 2002; Abdelaziz et al., 2023; Badamchi et al., 2017; Thi et al., 2020; Reboud et al., 2017; Hällgren et al., 2009
	ExoU, ExoS	PCR, ELISA (Li et al., 2005)	Contact cytotoxins	
	Exolysin ExlA (*exlA*)	ELISA (Deruelle et al., 2021)	Cytotoxicity	
	Siderophores: pyoverdine (*pvd*) and pyochelin (*pch*)	CAS-test (universal siderophore test) (Schwyn and Neilands, 1987)	Iron uptake (siderophore)	
	Pyocyanin overproduction (*phzA1B1C1D1E1F1G1-* and *phzA2B2C2D2E2F2G2*-operons, *phzS* and *phz*S)	ELISA (Pastells et al., 2016), extraction by chloroform and measuring the OD at 520 nm (Yang et al., 2012)	Cytotoxicity	
	Alginate hyperproduction (alginate operon, *algD*)	Congo red phenotypic test (Haddad et al., 2018), ELISA (Bryan et al., 1983), Carbazole assay (Wiens et al., 2014; Knutson and Jeanes, 1968)	Immune effector evasion and antibiotic resistance	
	Biofilm formation (*psl*, *pel*)	Christensen’s tube test (Christensen et al., 1982), biofilm ring test (Olivares et al., 2016), microtiter plate method (Peeters et al., 2008), Calgary biofilm device (Ceri et al., 1999)	Immune effector evasion and antibiotic resistance	
*E. faecium*	Cytolysin (*cyl*)	Hemolysis on Columbia agar base plates supplemented with (6%) horse blood (Hällgren et al., 2009), PCR (Vankerckhoven et al., 2004)	Cytotoxicity	Teng et al., 2003; Rathnayake et al., 2012; Semedo et al., 2003; Eaton and Gasson, 2002; Prasad and Jenq, 2024
	Surface protein (*esp*)	PCR (Vankerckhoven et al., 2004)	Adhesion, biofilm formation	
	Secreted antigen (*sagA*)	PCR (Teng et al., 2003)	Adhesion, biofilm formation	
*S. aureus*	Alpha hemolysin or alpha-toxin (*hla*)	Blood agar test (Mizobuchi et al., 1994), The *S. aureus* Alpha Hemolysin (Hla) ELISA Kit (Khodaverdian and Shoham, 2014), PCR (Tang et al., 2024)	Cytotoxicity	Hussain et al., 2022; Pinchuk et al., 2010; Divyakolu et al., 2019; Crosby et al., 2016; Shallcross et al., 2013; Peetermans et al., 2015; Bhattacharya and Horswill, 2024; Benson et al., 2011; Dinges et al., 2000; Zhu et al., 2023; Tang et al., 2019; Bukowski et al., 2010
	Aureolysin (*aur*)	Casein agar plate test (Bastock et al., 2021)	Tissue damage, cytotoxicity	
	Panton-valentine leucocidin (*pvl*)	CRISPR strip (Jin et al., 2024), PCR (Galia et al., 2019), ELISA, immunochromatographic test (Badiou et al., 2010)	Cytotoxicity, phagocytosis evasion	
	Toxic-shock syndrome toxin-1, or TSST-1 (*tstH*)	ELISA (Parsonnet et al., 1985), PCR (Schmitz et al., 1998; Abed et al., 2016)	Superantigen-induced shock, direct cytotoxicity	
	Enterotoxin (*sea-e*, *seh*, *sek*, *sem*, *sel*, *seo*, etc.)	ELISA (Rahimi, 2013), PCR (Schmitz et al., 1998)	Superantigen-induced tissue damage	
	Exfoliative toxins (*eta, etb, etd, ete*)	ELISA (Ladhani et al., 2001), PCR (Abed et al., 2016)	Damage to intercellular contacts as a result of desmosome cadherins hydrolysis	
	Clumping factor (*clfA* and *clfB*)	PCR (Ma et al., 2021), tube agglutination test (Crosby et al., 2021), latex agglutination test, slide agglutination test (Dickson and Marples, 1986), decrease in the plasma OD at 600 nm after incubation with bacteria (Crosby et al., 2021)	Biofilm formation	
	Coagulase activity: coagulase (*coa*) and von Willibrand factor-binding protein (vWbp)	Agarose plate coagulation assay (Bjerketorp et al., 2004), ELISA (Motta et al., 2023)	Immune effector evasion, biofilm formation	

*Focus is placed only on those methods that can be easily adapted to the clinical laboratory, with an emphasis on quantitative ones.

## Regulatory mechanisms

**FIGURE 2:**
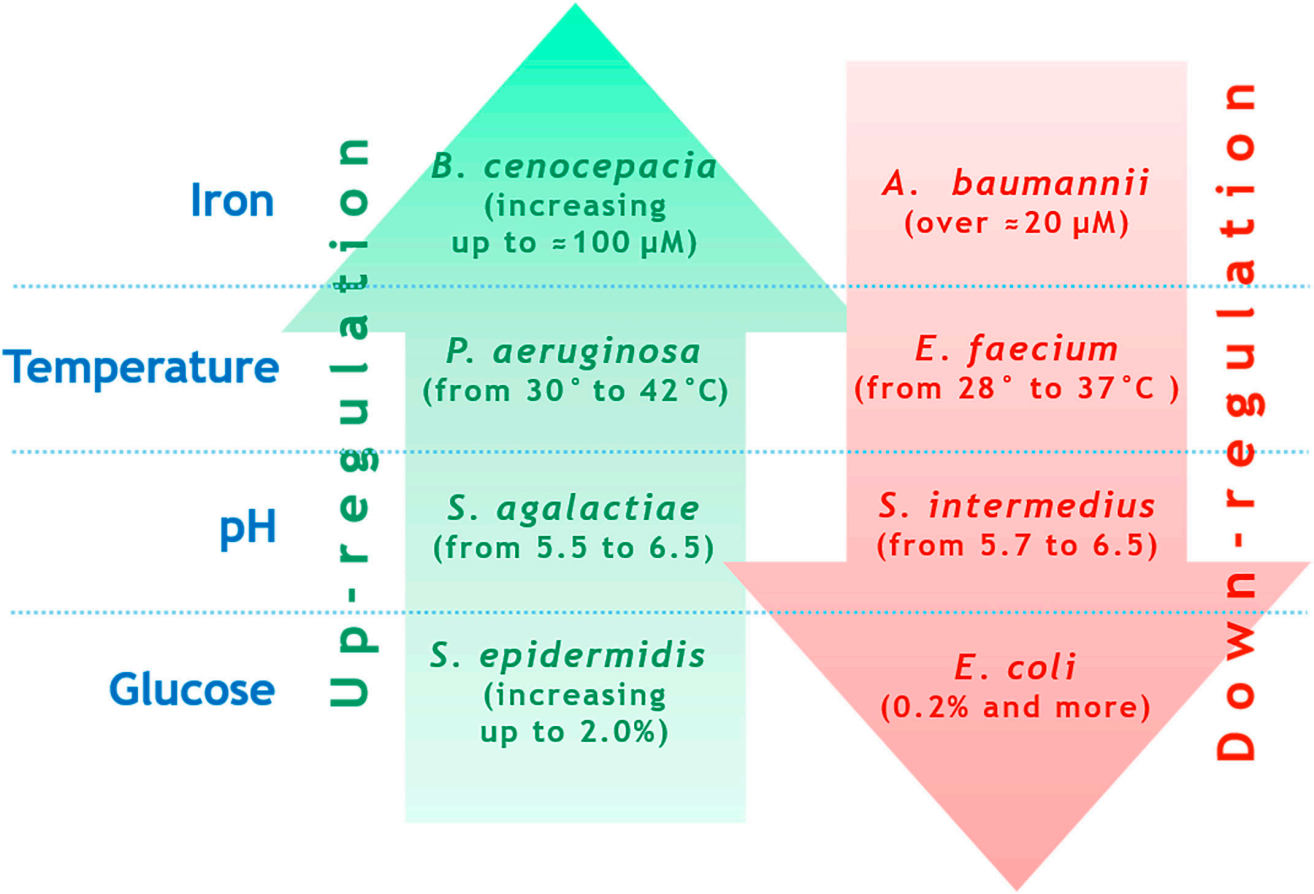
Examples of contrasting biofilm formation regulations for different species under changing external conditions (explanation is in the text).

Virulence factors are encoded by numerous genes, which are either scattered or organized into operons and clusters. The expression of virulence genes, post-translational assembly of their products, and in the case of some exotoxins - final activation inside the target cell - are complex processes controlled by global regulators and influenced by a variety of external conditions, including the microbial environment. Regulatory networks of virulence can vary significantly among different pathogens and have been extensively described in numerous reviews. While staying focused on the purpose of this review, we do not wish to repeat readily available summaries. However, we must emphasize the diverse ways in which external environmental factors influence virulence. For instance, biofilm formation intensity, which can be one manifestation of virulence, is highly sensitive to changes in cultivation conditions. Figure 2 demonstrates examples of how environmental factors affect biofilm formation in various opportunistic bacteria. Figure 2 does not claim to enumerate all possible scenarios but instead illustrates the divergent consequences of the same factors. Optimal Fe^3+^concentration for *K. pneumoniae* biofilm formation is approximately 50 μM, lowering Fe^3+^ concentrations significantly reduce biofilm formation (Chen et al., 2020). Optimal Fe^3+^ concentration for *Burkholderia cenocepacia* biofilm formation is roughly 100 μM, decreasing Fe^3+^ concentration reduces biofilm formation (Berlutti et al., 2005). Optimal Fe^3+^ concentration for *A. baumannii* biofilm formation is around 20 μM; increasing Fe^3+^ concentration drastically reduces biofilm formation (Modarresi et al., 2015). Increase in temperature enhances *P. aeruginosa* biofilm formation at 20 °C and 42 °C, with reduced formation between 25 °C and 30 °C (Grudniak et al., 2018; Kim et al., 2020). Conversely, *E. faecium* biofilm formation peaks at 28 °C compared to 37 °C (Maurya et al., 2021). The optimal pH for *S. aureus* biofilm formation is 7.0, deviations upward or downward reduce biofilm formation (Zmantar et al., 2010). Optimal pH for biofilm formation in *Streptococcus agalactiae* is 6.5, raising pH to 7.2 or lowering it to 5.5–4.2 diminishes biofilm formation (Miranda et al., 2018). Optimal pH for *Streptococcus intermedius* biofilm formation is pH 5.7, elevating pH to 6.5 significantly curtails biofilm formation (Ahmed et al., 2008). Addition of 0.2% glucose to growth media suppresses *E. coli* biofilm formation (Jackson et al., 2002). Meanwhile, *Staphylococcus epidermidis* biofilm formation increases with elevated glucose concentrations up to 1.5%, concentrations reaching 2.0% do not suppress biofilm formation (Rachid et al., 2000).

Biofilm formation is a function of the environment. It can be inferred that this rule applies to other virulence factors as well. For example, decreasing the level of environmental oxygen enhances the production of exotoxin A but reduces pyoverdin synthesis (Gaines et al., 2007). Hemolytic activity in *S. aureus* is significantly inhibited at 34 °C but realized at 37 °C (Bastock et al., 2021). Conversely, aureolysin production peaks at 34 °C and decreases by approximately 30% at 37 °C (Bastock et al., 2021).

These examples highlight the need for careful selection of standard conditions for phenotypic/biochemical assessments of virulence, ensuring that the production of virulence factors is not inhibited.

## Similarities, differences, and interference between the phenomena of virulence and antibiotic resistance

The main characteristics of antimicrobial resistance (AMR) and virulence have a lot in common (Table 2). They negatively impact the course and outcome of disease, can manifest as either constitutive (species-specific) traits or acquired features, and possess analogous principles of genetic regulation and dissemination mechanisms. Numerous instances have been described where enhanced virulence correlates positively with antimicrobial resistance. Genes coding for virulence factors and antibiotic resistance can be associated (Shah et al., 2019), including being co-localized within the same plasmid under unified control (Dong et al., 2022). Nonetheless, despite these commonalities, assessing resistance cannot substitute for evaluating virulence. This distinction arises because combinations of resistant and virulent properties in individual isolates exhibit considerable diversity, which manifests as classic interference in the physical sense of the term. The emergence of antibiotic resistance in a bacterial clone may coincide with decreased virulence, heightened virulence, or have no effect on virulence. The existence of all these variations has been illustrated in experiments involving uniform virulence evaluations of colistin-resistant *A. baumannii* strains. Colistin-resistant mutants with defects in the *lpx* gene, leading to complete loss of the polymyxin target lipopolysaccharide (LPS), exhibited diminished virulence in mouse and *Caenorhabditis elegans* experiments (Beceiro et al., 2014). In another study, colistin-resistant *A. baumannii* strains harboring mutations in *pmrB* (insertion of 30 nt or SNP 704C > T) did not diminish virulence toward *C. elegans* (Wand et al., 2015). An example of increased virulence in colistin-resistant *A. baumannii*, also confirmed using the *C. elegans* model, was associated with a mutation in the *pmrC* homolog *eptA*, a point mutation in ISAba1 upstream of *eptA*, and elevated *eptA* expression (Gerson et al., 2019). Presumably, the outcome of interference between AMR and virulence depends on (1) the chromosomal background of the isolate (its clonal lineage, inherent metabolic profile, baseline arsenal of virulence factors, etc.), (2) specific combinations of resistance and virulence determinants unique to every case, which may be dispersed or clustered as plasmid-borne genes, as well as mutations in chromosomal genes, (3) compensatory mutations that neutralize negative effects on the bacterium (disrupted functions) and partially or fully restore lost capacities arising from acquiring genetic determinants of virulence and resistance (Bæk et al., 2015). An important conclusion from this section is that the global properties of opportunistic pathogens’ virulence closely resemble those of AMR.

**TABLE 2:** Similarities and differences between antibiotic resistance and virulence.

Category	Antibiotic resistance	Virulence
Clinical significance	Negative: neutralization of the effect of antimicrobial therapy, deterioration of pathogen elimination, but no direct increase in tissue damage	Negative: tissue damage and deterioration of pathogen elimination
Main drivers	Indiscriminate use of antimicrobials (Allel et al., 2023)	Presence of a susceptible to the pathogen living organism (including the protozoa and insects), having an anti-infective defense (Casadevall and Pirofski, 1999; Gupta, 2023; Metz and Boldin, 2023)
Intrinsic (species-specific) property	Exists	Exists
Horizontal gene transfer (conjugation, transduction, transformation)	Exists (Koraimann, 2018; Wang et al., 2024; Hossain and Chowdhury, 2024; Lerminiaux and Cameron, 2019)	Exists (Koraimann, 2018; Penadés et al., 2015; Kelly et al., 2009; Griffith, 1928; Lin et al., 2016)
Possibility of occurrence/increase due to mutations in chromosomal genes	Exists (Doss, 1994)	Exists (Sokurenko et al., 1999)
Interplay with fitness	Does not affect, or increases, or decreases depending on AMR genetic mechanism and compensatory mutations (Beceiro et al., 2014; Wand et al., 2015; Gerson et al., 2019)	Increases fitness within the host (Cressler et al., 2016)
Positive interference with phage infection	Phages may contribute to AMR formation/spread due to transfer and spread antibiotic resistance genes (Muniesa et al., 2013)	Phages may contribute to increase/spread of virulence due to: (1) produce their own virulence factors (for example, Pf phage virions), (2) transfer and spread bacterial virulence genes, (3) directly determine bacterial virulence: virulence factor (including exotoxins) genes can be located in the genomes of prophages, (4) regulate expression of virulence genes not encoded by the phage: phage “moron” genes can increase virulence and fitness of host bacterium in human infection (Penadés et al., 2015; Spanier and Cleary, 1980; Waldor and Friedman, 2005; Boyd, 2012; Secor et al., 2020; Waldor, 1998; Taylor et al., 2019)

## Is the evaluation of virulence and fitness equivalent?

**FIGURE 3:**
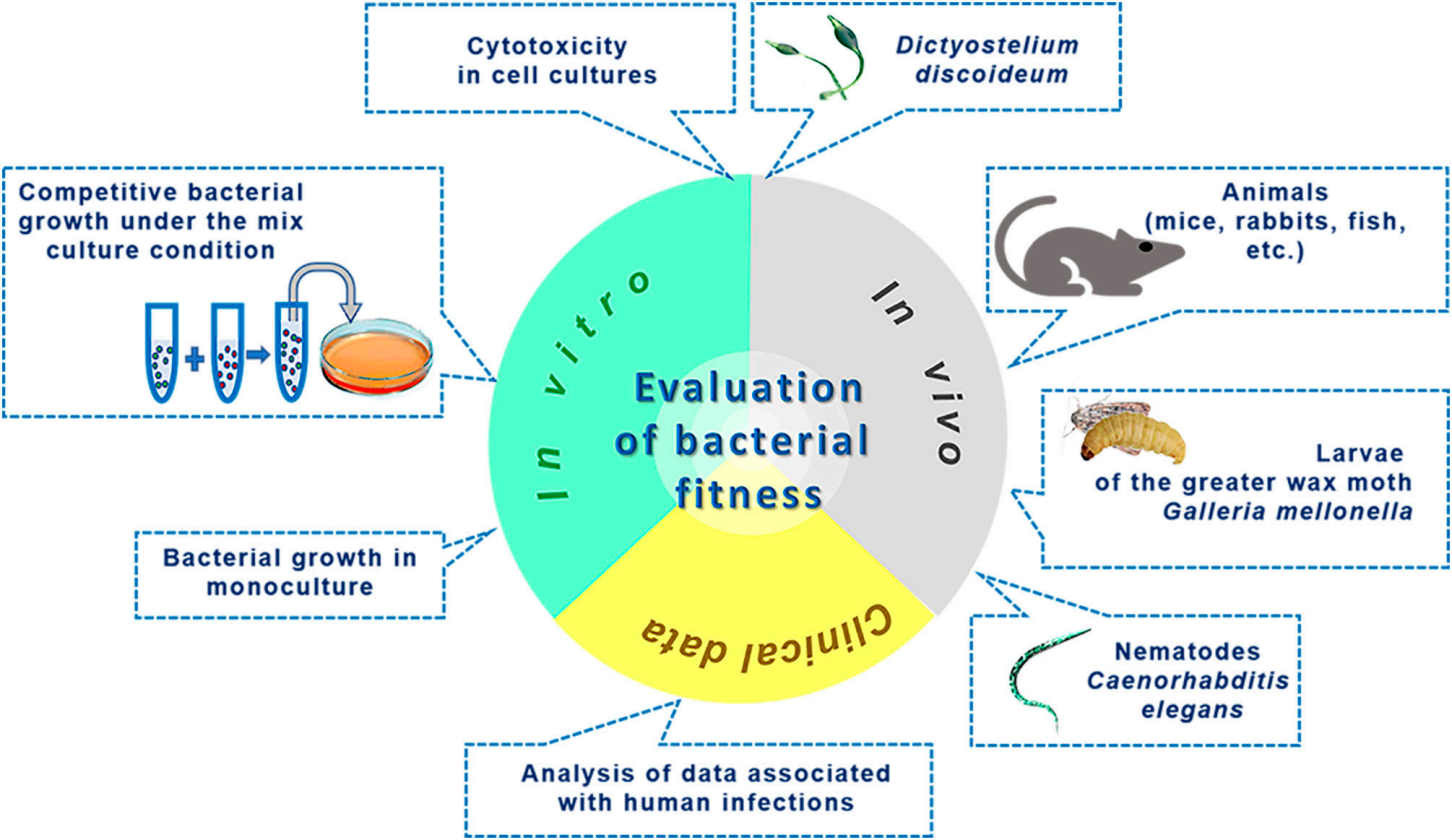
Methods to investigate microbial fitness.

When addressing the issue of bacterial fitness, we seek to understand whether the assessment of bacterial fitness is entirely identical to the evaluation of virulence. The roots of applying the term “fitness” in microbiology can be traced back to the work of Novick and Szilard (1950), wherein the authors modeled spontaneous mutagenesis and used the verb “to fit” (meaning “to correspond, to adapt”) in reference to mutants that evolved by acquiring properties better suited to changes in the nutrient medium than those of their ancestors (Novick and Szilard, 1950). Currently, there are several definitions of the concept of “fitness.” In a broader sense, it refers to the evolutionary success of an organism (Botelho et al., 2019). A more detailed interpretation describes fitness as the ability of a microbe to reproduce in a competitive environment under specific conditions dictated by physicochemical parameters of the microenvironment, nutrient availability, and the presence of antibiotics and other antimicrobial factors. This distinguishes fitness from virulence, which reflects the ability to inflict harm on the host organism. Numerous fitness evaluation methods often use reproduction times or replication speed under defined conditions as a basis (Yasui, 2022). The array of fitness assessment techniques is illustrated in Figure 3. *In vitro* methods, excluding cytotoxicity in cell cultures, rely on quantitatively determining bacterial survival and multiplication and do not entail evaluating the destructive potential of the pathogen, i.e., virulence. Methods for assessing fitness in animal models and cell culture systems may account for the integrative destructive impact on the object without specifying the activity of individual virulence factors. Additionally, animal models can characterize an essential element of the infectious process dependent on pathogen virulence - the ability to replicate within a living organism. Analyzing fitness using data related to infections is based on indirect integral outcomes. For instance, in the work of Boral et al. (2023), conclusions about fitness levels are drawn from analyzing disease outcomes. Although indicators such as proteolytic and lipolytic enzyme activities, efflux, iron exchange, toxin production, biofilm formation, or gene expression may sometimes correlate with fitness, they should not be equated with it completely, as this contradicts the definition of “fitness” outlined earlier. Instead, they can serve as good indicators of virulence.

Summarizing the content of this section, we conclude that the evaluation of fitness under *in vitro* conditions is not equivalent to the evaluation of virulence since it does not aim to determine the harm that the pathogen can inflict on the host. Meanwhile, studying fitness in *in vivo* models and cell cultures can yield useful insights into virulence, including the pivotal element of pathogenesis - the ability to multiply within live systems.

## Virulence in the context of bet-hedging

When discussing methodology choices for assessing virulence, it is essential to consider the bet-hedging strategy. Bet-hedging implies phenotypic and genetic heterogeneity within populations of microbial cells where different variants are adapted to different conditions (Veening et al., 2008). The aim of the bet-hedging is to increase fitness in temporally variable environment. In practical terms, this means that grown on artificial media subpopulations of the pathogen with the highest virulence may be suppressed. They may only be activated upon changes in the surrounding environment, such as invasion into the host organism (Byrne et al., 2025; Spratt and Lane, 2022). It stands to reason that upon invading the host, an opportunistic pathogen experiences stress due to pressure from the immune system. Under these circumstances, the microbe’s lifestyle undergoes a dramatic shift. Only those bacteria whose metabolism can sustain the infection process using virulence factors to achieve this, gain an advantage. Mechanisms of bet-hedging may vary: they occur through (1) shifts in dominant subpopulations within an initially heterogeneous population (Nuss et al., 2016) and/or (2) switching gene expression (Stewart and Cookson, 2012). Regardless of the mechanisms, under infection conditions the more virulent microbial population predominates. Therefore, the bacterial population within a patient may therefore differ substantially in its properties, including virulence, from populations grown on artificial media. This premise was proven in classical pneumococcal experiments dating back to the 19th century. Clinical and attenuated strains grown on artificial media increased their virulence within mice (Kruse and Pansini, 1892; Eyre and Washbourn, 1899; Wadsworth and Kirkbride, 1918). Had these researchers relied solely on the virulence assessment of pneumococci directly from artificial media to forecast their clinical hazard, they would have committed grave errors.

From these observations emerges a vital insight, resonant with the conclusion drawn earlier: when designing systems for cultivating bacteria to evaluate their virulence phenotypically, conditions should not be optimized for the best growth and proliferation of a species. Instead, conditions fostering maximal production of virulence factors should be prioritized.

## Methods for virulence evaluation

*In vivo* evaluation is the gold-standard method for testing virulence, reflecting the integrated interaction between the host and the microbe. The significance of each virulence factor should ideally be confirmed through animal studies and clinical observations. Exceptions may only be made for close orthologs of already tested factors in closely related species or intraspecific variants. Unfortunately, *in vivo* virulence evaluation is not possible in routine clinical practice, which requires alternative testing methods.

While identifying individual key virulence factors does not equate to the comprehensive *in vivo* evaluation of virulence, the combination of these key factors (the “virulence puzzle”) can correlate with *in vivo* assessments. This assertion is supported by various experimental findings (Pimenta et al., 2006; Wikraiphat et al., 2009; Lavender et al., 2004; Williamson et al., 2014).

For virulence evaluation methods to be effective as monitoring tools, they must meet the following requirements:

1. Be technologically and financially accessible to all laboratories performing routine clinical microbiology tests.
2. Provide results within a reasonably short timeframe, ideally not exceeding the duration of phenotypic AMR testing.
3. Yield consistently reproducible results on reference strains.
4. Accurately and linearly measure acceptable quantitative fluctuations in the activity of specific virulence factors that are key factors to the tested species.
5. Have optimal sensitivity and specificity in detecting virulence factor.
6. Generate digitized results suitable for statistical analysis.
7. Employ a comprehensive approach, encompassing the determination of a set of virulence factors for each species, where the cumulative index or score correlates with the level of virulence verified *in vivo* or based on clinical data.
8. Ensure safety by avoiding the use of live organisms, such as laboratory animals.

At first glance, whole genome sequencing (WGS) appears ideal for virulence assessment (Wolff et al., 2021). However, its application as a monitoring tool faces challenges. First, the ambiguous role of numerous genes and mutations in virulence realization complicates interpretation. Second, the complexity, expense, and prolonged execution render WGS inaccessible to many laboratories. Although sequencing cost has decreased by a factor of 1,000 over the past 20 years, it still remains too high for routine use in clinical laboratory practices. The cost of raw data (“reads”) of whole genome sequencing (WGS) varies depending on local conditions and study volume, ranging from US$40 to US$100 per bacterial genome. The minimum time required from DNA isolation to obtaining raw sequencing data (assuming automatic library preparation and maximum sequencer loading) is at least 3 days. Another reason hindering personalized utilization of WGS in virulence assessment is the imperfection of bioinformatic interpretation of virulome data. We are confident that in the future, accessible sequencing technologies and advanced automated bioinformatic tools will become available, enabling reliable information retrieval on the virulence of each clinical isolate. Currently, practical feasibility dictates focusing on identifying a limited number of virulence factors with proven pathogenic relevance, capable of enhancing the baseline intrinsic virulence level inherent to members of a given species.

Polymerase chain reaction (PCR)-based methods excel in detecting individual resistance genes, as shown in Table 1. However, their inability to quantify virulence factor production limits their utility for virulence detection. Similarly, newer pathogenicity testing methods based on CRISPR strips (Jin et al., 2024) suffer from the same limitations as PCR.

Proteomic techniques, such as mass spectrometry, hold great promise for future applications in virulence assessment (Man et al., 2021; Flores-Trevino et al., 2019). Specifically, encouraging results have been achieved using MALDI-TOF MS to identify hypervirulent variants of *Clostridioides* (*Clostridium*) *difficile* (Flores-Trevino et al., 2019; Godmer et al., 2024). Unfortunately, the current application of MS for virulence monitoring is limited by its inability to quantitatively determine virulence factor production by direct profiling without complex and time-consuming sample preparation. Consequently, mass spectrometry currently fails to meet the aforementioned requirements for virulence monitoring.

Below, we discuss methodological approaches that could serve as a basis for detection of virulence and have demonstrated successful usage.

**FIGURE 4:**
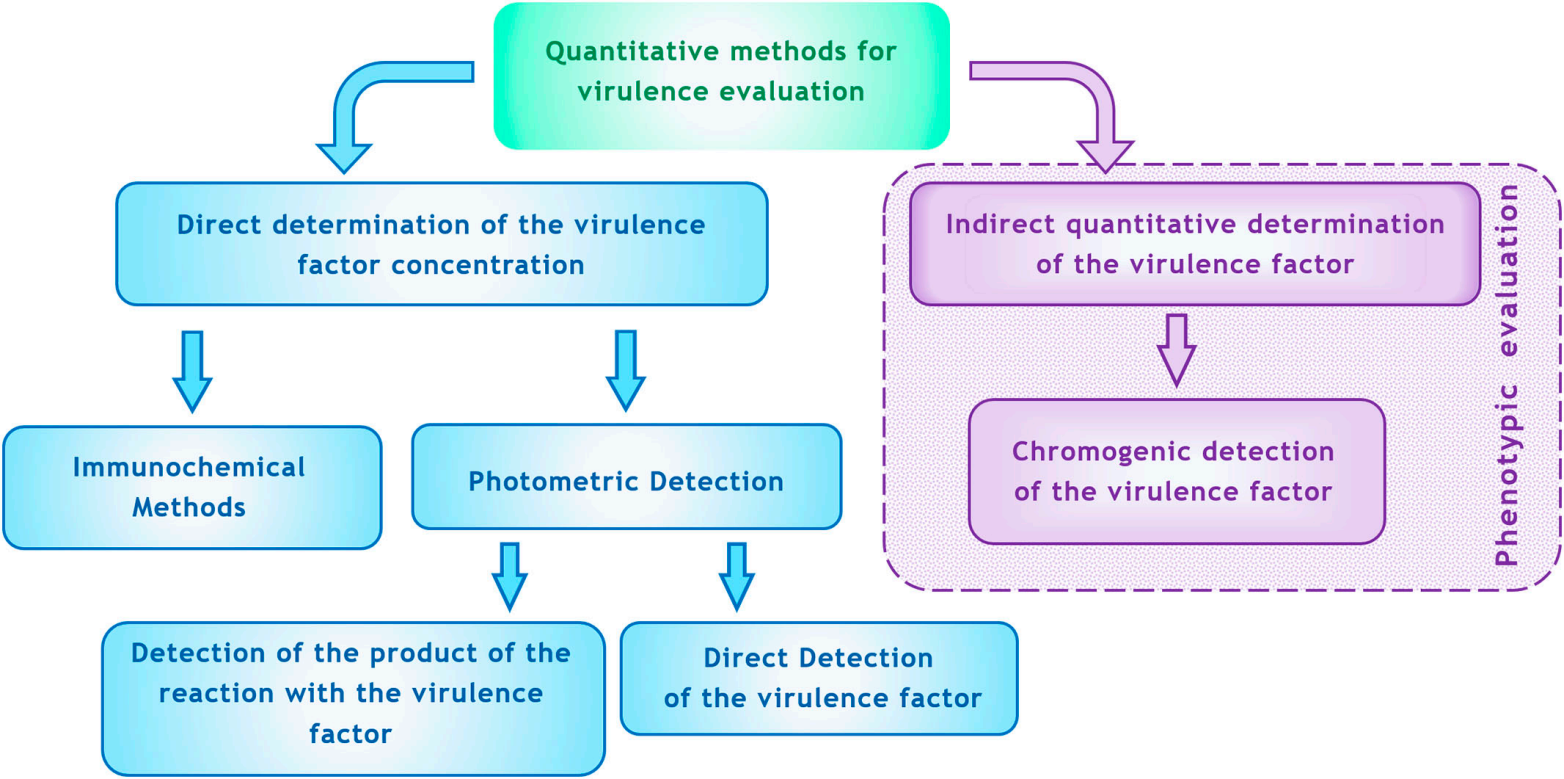
Methods for quantitative determination of virulence factors *in vitro* (explanations are in the text).

For clinical microbiology practices, paralleling resistance testing, two options for quantitatively assessing virulence are convenient: (1) direct determination with precise measurement of the concentration of the virulence factor, and (2) indirect determination with quantitative metrics correlated with the level of virulence but lacking exact concentration measurements. Potential methods for detection of virulence are depicted in Figure 4.

As a direct method for determining virulence factors, immunochemical techniques can be employed. These methods have a long history. Serological methods for detecting antigenic structures associated with exotoxins (such as diphtheria toxin, botulinum toxin, etc.), LPS (O-antigen), flagella (H-antigen), capsules (e.g., for serotyping *S. pneumoniae*), and streptococcal M-antigens have become routine. Historically, these methods focused on qualitative determination of toxigenicity or taxonomic classification (species, serogroups, serotypes). More recently, immunoassays have been developed to quantitatively measure virulence factors. For instance, ELISA has successfully been used to assess virulence factors in *S. aureus*, enabling the quantitative determination of staphyllococcal enterotoxin (SE) (Rahimi, 2013), alpha-hemolysin (Kehoe et al., 1983), toxic shock syndrome toxin-1 (TSST-1) (Parsonnet et al., 1985), protein A (Löfdahl et al., 1983), exfoliative toxin (ET) (Ladhani et al., 2001), and von Willebrand factor-binding protein (vWbp) (Motta et al., 2023). Immunoassays have also proved effective for quantitatively measuring *P. aeruginosa* virulence factors, including pyocyanin and 1-hydroxyphenazine (Pastells et al., 2016), alkaline proteinase, elastase, and exotoxin A (Shigematsu et al., 2007), and exolysin ExlA (Deruelle et al., 2021) using ELISA.

Moreover, commercial kits based on immunoassays are available not just for research purposes. For example, Cusabio Biotech Co., Ltd. (Hubei, China) manufactures a commercial Human Pseudomonas Exotoxin A PEA ELISA Kit for detecting *P. aeruginosa* exotoxin A. Oxoid Ltd. (Hampshire, United Kingdom) produces sets for detecting exotoxins, including the ProSpecT™ STEC (Shiga Toxin *E. coli*) Microplate Assay, ProSpecT™ *C. difficile* Toxin A/B Microplate Assay, SET-RPLA Kit Toxin Detection Kit for semi-quantitative detection of staphylococcal enterotoxins A, B, C, and D, and TST-RPLA Toxin Detection Kit for semi-quantitative detection of staphylococcal toxic shock syndrome toxin. Abcam Ltd. (Cambridge, United Kingdom) offers Protein A ELISA Kits for the quantitative determination of *S. aureus* protein A. These developments suggest that modern technologies and manufacturing capabilities can facilitate global monitoring of virulence among opportunistic pathogens using direct quantitative immunoassays.

Despite their advantages, immunoassays face certain limitations. They cannot detect virulence factors composed of simple small molecules (e.g., cyanides produced by some glucose-non-fermenting Gram-negative bacteria) (Enderby et al., 2009) or molecules exhibiting substantial variability within a single species (e.g., *P. aeruginosa* strains produce around 60 variants of rhamnolipids) (Abdel-Mawgoud et al., 2010).

Another category of methods relies on direct optical measurements to determine the concentration of virulence factors. Table 3 summarizes known methods for quantitatively assessing virulence factors using spectrophotometry. Spectrophotometric techniques have been applied to (1) measure reaction outcomes where a virulence factor exhibits enzymatic properties, (2) directly quantify virulence factors based on their intrinsic optical characteristics (e.g., pyocyanin), and (3) detect virulence factors by staining them with specific dyes (e.g., siderophores, rhamnolipids, cyanide, biofilm matrix, alginate, LPS). Notably, some of these methods have been incorporated into commercially available kits, such as SideroTec™ Total Assay Kits (Accuplex Diagnostics Ltd., Kildare, Ireland) for siderophore determination, Thermo Scientific™ Pierce™ Colorimetric Protease Assay Kit (Thermo Fisher Scientific Inc., MA, United States) for protease detection, Lipase Activity Assay Kit (Abcam Ltd., Cambridge, United Kingdom) for lipase detection, and ToxinSensor™ Chromogenic LAL Endotoxin Assay Kit (GenScript Biotech Corporation, NJ, United States) for endotoxin (LPS) detection.

**TABLE 3:** Known spectrophotometric methods for quantitative determination of some key virulence factors.

Virulence factor	Bacteria	Method	References
Alginate	*P. aeruginosa*	Carbazole assay based on uronic acid determination, measuring the OD at 530 nm	Knutson and Jeanes, 1968; Wiens et al., 2014
Alpha-hemolysin	*S. aureus*	Spectrophotometric determination of rabbit erythrocyte hemolysis by measuring optical density (OD) at 541 nm	Marucci, 1963; Ridder et al., 2021
Biofilm matrix	*A. baumannii*, *P. aeruginosa*, *S. aureus*, *K. pneumoniae*, etc.,	Staining with safranin, measuring the OD at 530 nm or Staining with crystal violet, measuring the OD at 595 nm	Silva et al., 2021; Toledo-Arana et al., 2001
Catalase	*A. baumannii*	The Catalase Colorimetric Activity Kit	Sato et al., 2019
Clumping factor	*S. aureus*	Decrease in the plasma OD at 600 nm after incubation with bacteria	Crosby et al., 2021
Phospholipase D	*A. baumannii*	Phospholipase D (PLD) Activity Colorimetric Assay Kit	Elabscience, 2025
Pyocyanin	*P. aeruginosa*	Extracted by chloroform and measuring the OD at 520 nm	Yang et al., 2012
Total siderophores	*A. baumannii*, *P. aeruginosa*, *K. pneumoniae*, *E. coli*, etc.,	Staining with Chrome azurol S (CAS), measuring the OD at 530 nm	Schwyn and Neilands, 1987

While spectrophotometric methods based on direct *in vitro* determination of virulence factors offer many benefits, they have yet to be applied to the detection of exotoxins. This limitation is easily explained. Many exotoxins only display their enzymatic properties after undergoing final modifications within the target cell. For instance, *P. aeruginosa* exotoxin A must undergo disulfide bond reduction facilitated by disulfide isomerase, which occurs in the early endosomes of eukaryotic target cells, before acquiring its full toxic properties as an NAD^+^-diphtamide-ADP-ribosyltransferase (Michalska and Wolf, 2015; Krueger and Barbieri, 1995). Contact toxins operate via a distinct activation mechanism within eukaryotic cells. For example, *P. aeruginosa* ExoU must be ubiquitinated to acquire its toxic and enzymatic attributes (Foulkes et al., 2019). Superantigens generally lack enzymatic activity, making their detection through spectrophotometric reactions challenging.

Nevertheless, we believe that detecting exotoxins via photometric methods is feasible. To accomplish this, it is necessary to recreate the conditions for toxin activation *in vitro* and subsequently measure the enzymatic activity of the toxin in a reaction with a substrate. No theoretical barriers prevent this approach. In fact, the history of ExoU investigation demonstrates that restoring ExoU toxicity *in vitro* has already been achieved. Sato et al. (2006) treated pro-toxin ExoU with superoxide dismutase, after which it gained toxic properties. While the authors concluded that superoxide dismutase acted as an obligate cofactor for ExoU, subsequent studies revealed that ubiquitination is the true driver behind ExoU activation (Stirling et al., 2006). The observed activation by superoxide dismutase likely resulted from insufficient purification of the enzyme from ubiquitin. Fortunately, this methodological error holds immense positive implications: it proves that exotoxin activity can indeed be restored *in vitro*, thereby opening doors for novel detection methods. In, Zhang et al. (2017) corroborated this idea by demonstrating that treating ExoU with phosphatidylinositol 4,5-bisphosphate enabled it to acquire phospholipase activity and thus toxicity.

Another intriguing and highly promising approach for detecting virulence factors involves the use of chromogenic substrates. Within the scope of this review, we define “chromogenic substance” as a compound that can be cleaved by specific enzymes at predefined locations, resulting in a visible color change. Chromogenic substrates boast several notable qualities: (1) high substrate specificity when interacting with a particular enzyme, (2) versatility, as there are no theoretical constraints preventing the design of custom chromogens for virtually any enzyme, and (3) cost-effectiveness and ease of interpreting reaction outcomes.

These attributes position chromogenic substrates as cornerstones for building quantitative assessments of virulence factors. Chromogenic virulence testing can be performed on agar-based media. Previous experience with semiquantitative evaluations of virulence factors on agar plates enriched with special additives - whereby the virulence factor generates a colorless or colored halo around the bacterial colony - provides a solid foundation. The diameter of this halo correlates with the quantity of the virulence factor. One of the most well-known examples is the determination of hemolysins on blood agar. Bacterial isolates are plated on sheep blood agar plates, and after incubation, the diameter of the hemolysis zone around the colonies is measured (Stulik et al., 2014; Sankomkai et al., 2020). Using this technique, key virulence factors such as *S. aureus* α-toxin and *E. faecium* cytolysin have been assessed (Hällgren et al., 2009; Mizobuchi et al., 1994). *S. aureus* aureolysin was analyzed using TSB agar supplemented with 15% non-fat milk (Bastock et al., 2021). *S. aureus* coagulase activity was determined on agarose plates (0.9%) containing bovine fibrinogen and 1% plasma (Bjerketorp et al., 2004). For lipase detection, triglycerides (e.g., tributyrin) are added to agarized agar, and results are evaluated by measuring the clear zone diameters around colonies (Sankomkai et al., 2020). DNAse activity is measured on DNA-agar or DNA-agar-Toluidine Blue-O dye diffusion systems. After bacterial incubation, the agar surface is treated with HCl, and the diameter of the resultant clearing zone around colonies correlates with DNAse activity (Sankomkai et al., 2020). In the TDA system, a pink zone of activity around colonies is measured (Kamman and Tatini, 1977). To assess extracellular protease production, bacterial suspensions are plated onto casein agar plates incorporating bromocresol green dye. The transparent zone of protease activity around colonies reflects protease activity (Vijayaraghavan and Vincent, 2013). For qualitative analysis of siderophore production, the Chrome Azurol Sulfonate (CAS) agar plate assay is employed. Here, the release of CAS dye from the Fe^3+^-dye complex causes a color change from blue to orange, signifying siderophore production (Kronda et al., 2013). There are numerous variations of these methods, differing in technical details and dyes. The underlying principle remains constant measuring the diameter of the virulence factor diffusion zone in agar. It’s worth noting the existing precedent for qualitatively determining virulence factors based on specific enzymatic properties using chromogenic agar. CHROMagar has introduced CHROMagar™ STEC for detecting Shiga-Toxin-producing *E. coli* (Gouali et al., 2013). This medium exemplifies how a chromogenic substance can act as an enzyme target, the presence of which correlates with the production of virulence factors (in this case, Shiga toxin). This underscores the potential of chromogenic testing to reflect the production of virulence factors, even those lacking enzymatic activity. This is especially pertinent for detecting superantigens, which are not enzymes.

In our view, combining virulence factor diffusion with chromogenic indication presents a promising pathway for advancing virulence factor determination, analogous to the disk-diffusion method for AMR testing. Implementation of such methods for quantitative virulence testing will require standardizing bacterial quantities. This task is technically achievable using auxiliary devices, such as membrane micro-inserts, which limit bacterial spread during incubation. Another standardization option could involve basing results not on the diameter of color changes in agar around the colony but on the ratio between the area of altered-color agar and the colony diameter.

The merits of employing chromogenic agars for virulence detection include technological accessibility, relative cost-effectiveness, ergonomics, and user-friendly result recording.

**FIGURE 5:**
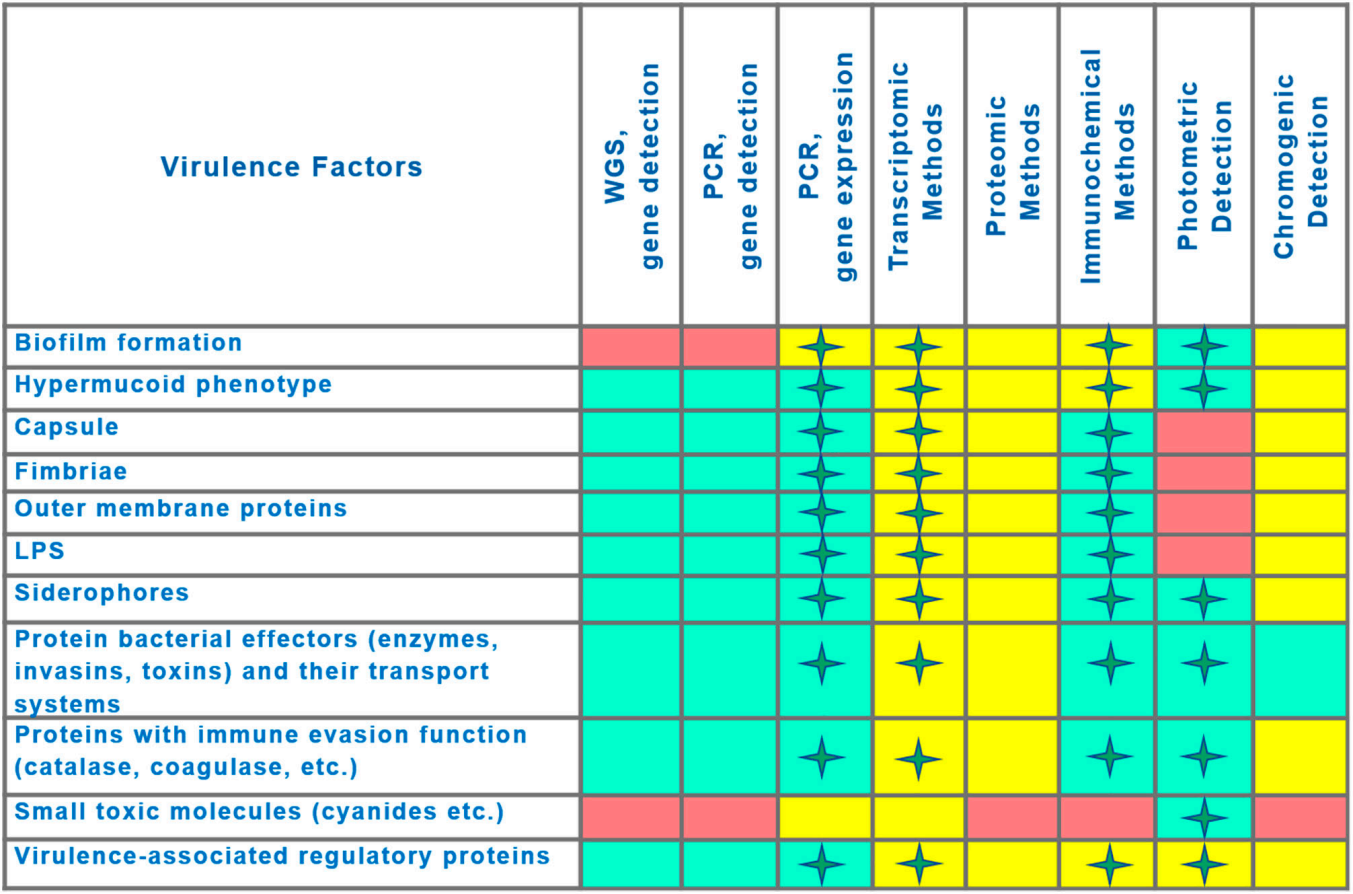
Comparison of capabilities of methods for virulence detection. Asterisks indicate the possibility of quantitative measurement of virulence factor production. Capability of method to detect the virulence factor is indicated by the rectangle fill color as follows: Green, application is feasible; Pink, application is not feasible; Yellow, capabilities have been demonstrated, but practical applicability at present is uncertain (technological or financial limitations).

A visualization of the comparison of virulence detection methods is presented in Figure 5. Likely, the decisive factor in choosing a detection method will be determined by the balance between result quality (quality criteria described above) and accessibility for routine practice (Supplementary Table 2).

## Future directions

Key prerequisites for implementing virulence detection in clinical practice include method standardization/validation and executing a pilot project. Initial implementation steps could comprise sequential actions including (1) selecting, adapting, and standardizing optimal clinically-relevant methods for detecting virulence factors in several relevant opportunistic pathogens (e.g., ESKAPE group) based on literature data, (2) attracting funding from non-profit foundations and interested businesses to support pilot studies in a limited number of clinical laboratories, (3) conducting pilot studies in select clinical labs, (4) collecting, analyzing, and publishing/reporting collected data on the pathogenic role of virulence factors to enhance awareness and understanding of their clinical significance. If positive results are achieved, it would be possible to initiate broader cooperation with interested public organizations (ESCMID research groups, etc.), and manufacturers of diagnostic kits. Our review aligns with the first point of this action plan, and we expect it to serve as a stimulus increasing interest among professional organizations and their leaders in addressing the issue of virulence.

## Limitations

Our review has several limitations. Firstly, we analyzed the virulence of opportunistic pathogens, representing just one side of the infection process, without considering the host immune system’s contribution, the impact of microbiota, or comorbidities. Secondly, the review focused primarily on a limited number of opportunistic bacterial species, for which fewer controversies exist in the literature regarding their pathogenic factors. The existing imbalance in the amount of controversy may stem from unequal research intensity directed toward different species and the disparity in the number of published works dedicated to various species. Additionally, the incomplete state of knowledge regarding genetic determinants of virulence in opportunistic pathogens restricts the perfect interpretation of virulence, although it does not preclude the detection of known virulence factors for informational purposes. Lastly, the review did not incorporate an analysis of correlations between virulence factors and the transmissibility of the infectious agent.

## Conclusion

Current technological advancements now enable the integration of virulence detection for main opportunistic pathogens into clinical laboratory practice. To briefly justify the necessity of introducing virulence testing into clinical routines, it is imperative to address two central questions. What benefits will virulence detection bring to individual clinical cases? And what advantages will global-level monitoring of virulence offer? Answers to these queries exist:

1. Determining the virulence of isolates from individual patients improves the prediction of the progression of infectious processes. The activity of virulence factors can mirror the actual threat posed by a pathogen, shedding light on the likelihood of infection chronicity or exacerbation.
2. Evaluating the virulence profiles of circulating nosocomial pathogens aids in rationalizing infection control at the hospital level, drawing on principles similar to those guiding the fight against antibiotic-resistant strains.
3. As virulence testing becomes more widespread, data on the global prevalence of highly virulent strains will accumulate, facilitating optimization of anti-epidemic measures and enabling healthcare resources to be reallocated strategically toward the greatest threats.
4. Finally, monitoring virulence may prove invaluable for informing strategies to develop antivirulence therapy.
