# Supplementary material for: Detection of virulence factors in opportunistic bacteria: advances, challenges, and practical implementation
Source: Front Microbiol. 2025 Sep 17;16:1638925. doi: 10.3389/fmicb.2025.1638925 (PMC12484208; doi:10.3389/fmicb.2025.1638925)
Supplement: Supplementary file 1 [file Table_1.docx]

**The Supplementary Materials**

**Table S1.** Glossary: definitions of the key terms used in the review

| No | Term | Definition |
| --- | --- | --- |
|  | Acquired virulence factor | The virulence factor with high level of variability among strains of the same species |
|  | Bet-hedging | The phenotypic and genetic heterogeneity within populations of microbial cells where different variants are adapted to different conditions; the aim of the bet-hedging is to increase fitness in temporally variable environment. |
|  | Fitness | The capacity of an individual or genotype to survive and reproduce under specific environmental conditions. |
|  | Key virulence factor | The virulence factor without which opportunistic pathogens become incapable of causing disease or result in mild forms of the disease. The concept of the key virulence factor can include both factors encoded by a single gene and factors whose production may depend on a set of different genes. Perhaps the term “key factor” will be more convenient for the phenotypic assessment of virulence. |
|  | Opportunistic pathogens | Microorganisms that in contact with the human body are cause disease only in exceptional cases; opportunistic pathogen is said to be a microbe that can cause disease only when the host’s resistance is altered |
|  | Pathogens (non-opportunistic) | Microorganisms that are cause disease in practically healthy persons with high probability. Practically healthy persons are persons in whom pathological changes observed in the body do not affect the subjective state of being and are not reflected in the anti-infective resistance and person's efficiency. |
|  | Pathotype (pathovar, virotype, virulotype) | 1. A disease-causing variant of a microorganism. It is distinguishable from other members of its species by its virulent properties and by unique molecular markers. 2. A taxonomic category below the species level, identified by shared features of disease-causing ability, specifically with regard to the range of hosts affected. 3. An intra-species group of microorganisms with the same set of virulence genes.  A cross-pathovar is a strain that possesses recognised hallmarks of several pathovars. |
|  | Virulence | The degree of the injury-producing potential or toxicity of a microorganism. |
|  | Virulence factor | The virulence factor is a component of a pathogen that contributes to the progression of the infectious process; virulence factors can include modulins for host immune effectors. |
|  | Virulence index | The sum of all positive virulence phenotype exhibited by the isolate (none weighted) divided by total number of virulence factor tested. |
|  | Virulence score | The sum of the virulence factor genes in each isolate. |
|  | Virulome | The set of genes that contribute to the virulence of a pathogen. Virulome includes (1) true virulence genes, that are directly involved in causing disease, (2) virulence-associated genes that encode regulators/activators of true virulence genes, (3) virulent life-style genes that ensure viability in the host organism. |
