# Supplementary material for: Detection of virulence factors in opportunistic bacteria: advances, challenges, and practical implementation
Source: Front Microbiol. 2025 Sep 17;16:1638925. doi: 10.3389/fmicb.2025.1638925 (PMC12484208; doi:10.3389/fmicb.2025.1638925)
Supplement: Supplementary file 2 [file Table_2.docx]

**The Supplementary Materials**

**Table S2.** Examples of cost/accessibility of some tests that can be used for assessing virulence factors

| Methods | | Cost per isolate/test, US$ * | Technological feasibility in routine clinical practice | Availability in resource-limited settings |
| --- | --- | --- | --- | --- |
| Genetic/genomic | WGS | 40.0 – >100.0 | Infeasible | Unavailable |
|  | PCR and other Amplification-based Methods | ~ 1.0 – 17.0 | Feasible | Partially available |
| Immunochemical | ELISA (Staphylococcus aureus enterotoxin ELISA Kit, Human Pseudomonas Exotoxin A ELISA Kit, etc.) | 2.5 – 20.0 | Feasible | Partially available |
| Phenotypic | Lipase, protease, DNAse, haemolysins | 1.2 – 4.5 | Feasible | Available |
|  | Chromogenic | ≥ 1.0 | Feasible | Available |
| Photometric | Siderophores detection (SideroTec™-Total Assay Kit) | ~ 3.0 - 4.0 | Feasible | Partially available |
|  | Enzymes (Colorimetric Protease Assay Kit) | ~ 2.0 | Feasible | Available |
| * The data was obtained from websites https://www.elabscience.com, https://www.molv.org, https://accuplexdiagnostics.com, https://www.thermofisher.com, https://hardydiagnostics.com, https://sequencing.com  Data depend on region/country and local characteristics | | | | |
